# The application of ERAS Protocol in Laparoscopic Radical Cystectomy combined with Cutaneous Ureterostomy and its impact on postoperative recovery

**DOI:** 10.12669/pjms.41.8.12310

**Published:** 2025-08

**Authors:** Min Lei, Weiwei Cao, Tingting Chao, Longhua Lu, Hongying Fan, Kun Liu

**Affiliations:** 1 Min Lei Department of Urology Surgery, West China Xiamen Hospital of Sichuan University, Xiamen, Fujian Province 361022, P.R. China; 2 Weiwei Cao Department of Neurological Rehabilitation, Xiamen Humanity Rehabilitation Hospital, Xiamen, Fujian Province 361015, P.R. China; 3 Tingting Chao Department of Chest Disease Center, West China Xiamen Hospital of Sichuan University, Xiamen, Fujian Province 361022, P.R. China; 4 Longhua Lu Department of Urology Surgery, West China Xiamen Hospital of Sichuan University, Xiamen, Fujian Province 361022, P.R. China; 5 Hongying Fan Department of Head and Neck Oncology, West China Hospital of Sichuan University, Chengdu, Sichuan Province 610041, P.R. China; 6 Kun Liu Department of Urology Surgery, Xiamen Humanity Hospital Fujian Medical University, Xiamen, Fujian Province 361015, P.R. China

**Keywords:** Cutaneous ureterostomy, Complications, Enhanced Recovery After Surgery, Laparoscopic radical cystectomy

## Abstract

**Objective:**

The effectiveness of the Enhanced Recovery After Surgery (ERAS) protocol in patients undergoing laparoscopic radical cystectomy combined with cutaneous ureterostomy (LRC-CU) is unclear. This study aimed to explore the effectiveness of Enhanced Recovery After Surgery (ERAS) protocols in patients undergoing laparoscopic radical cystectomy combined with cutaneous ureterostomy (LRC-CU).

**Methods:**

This retrospective study included clinical records of patients who underwent LRC-CU in the West China Hospital of Sichuan University from October 2022 to October 2024. Patients were divided into the ERAS and the non-ERAS groups based on the adopted perioperative protocol. Postoperative recovery status, the Herth Hope Index (HHI), the Assessment of Cancer Therapy-General (FACT-G), and the incidence of complications were assessed.

**Results:**

A total of 105 patients were included, with 54 patients in the ERAS and 51 in the non-ERAS group. The times to postoperative exhaustion, first bowel movement, getting out of bed, and length of hospital stay were significantly shorter in the non-ERAS group (P < 0.05). After surgery, the HII and FACT-G scores in both groups increased compared to preoperative values and were significantly higher in the ERAS group compared to the non-ERAS group (P < 0.05). The incidence of complications in the ERAS group (3.70%) was lower than that in the non-ERAS group (17.65%) (P<0.05).

**Conclusions:**

The ERAS protocols adopted by LRC-CU can significantly shorten the rehabilitation process, reduce the incidence of complications, and improve the HHI and the quality of life (QOL) of LRC-CU patients.

## INTRODUCTION

Bladder cancer is caused by factors such as hereditary predisposition, chronic infection, smoking, etc., with the incidence ranking first among the malignant tumors of the urinary system.^1^–^3^ At present, radical cystectomy (RC) combined with various urinary diversion surgeries (UDS) is the standard surgical method for the treatment of muscle-invasive bladder cancer.^1^,^3^ Laparoscopic radical cystectomy combined with cutaneous ureterostomy (LRC-CU) has the advantages of simple operation, minimal trauma, low postoperative bleeding, and relatively low risk of complications such as infection.^4^,^5^ However, UDS are associated with a lifelong requirement for urine bags, which profoundly impacts the quality of life of patients.^3^–^5^ Therefore, implementing effective nursing interventions after surgery is of great significance.

In recent years, the Enhanced Recovery After Surgery (ERAS) protocols have been widely adopted in urological surgeries, such as radical cystectomy (RC).^6^,^7^ As ERAS emphasizes interdisciplinary collaboration and adopts a series of evidence-based optimization measures during the perioperative period, it can alleviate psychological and physiological traumatic stress reactions, reduce complications and mortality, shorten hospitalization time, and lower the risk of readmission and associated healthcare costs.^3^,^6^–^9^

Nevertheless, there is still limited evidence to support the application of ERAS protocols in patients after LRC-CU.^3^,^6^,^7^ This study aimed to explore the application effect of ERAS protocols in the perioperative period of individuals undergoing LRC-CU, providing a reference for rehabilitation nursing in this patient group.

## METHODS

This single-center retrospective analysis was conducted at the Department of Urology, West China Hospital, Sichuan University. The data was selected from patients who underwent LRC-CU between October 2022 to October 2024. Patients were divided into the ERAS group and the non-ERAS group based on whether they received ERAS during the perioperative period.

### Ethical Approval:

The study was approved by the ethics committee of West China Xiamen Hospital of Sichuan University approved this study, approval number: 2024(084); dated: November 25, 2024.

### Inclusion criteria:

- Conforming to the diagnostic criteria of bladder cancer and confirmed by pathological examination.^1^
- Age range from 18 to 75 years.
- Complete clinical data.

### Exclusion criteria:

- Patients with mental illness, inability to communicate normally, or inability to care for themselves—defined as those with severe physical or cognitive impairments that prevent them from performing basic daily activities such as feeding, dressing, or mobilizing without assistance—were excluded.
- Patients with coagulation dysfunction.
- Patients with other malignant tumors.
- Patients with previous intestinal surgery and severe heart/liver/lung/kidney dysfunction.
- Detailed information on the key perioperative management steps for the ERAS and non-ERAS groups is presented in Table-I. The non-ERAS group followed a more conservative postoperative care protocol, which included routine pain management, delayed mobilization, and a gradual approach to nutrition and early mobilization. Patients were encouraged to rest in bed for the first 48 hours after surgery, with gradual increases in activity as tolerated. The focus was on minimizing immediate postoperative stress. All operations were performed by the surgical team, which has over eight years of experience in LRC-CU procedures. Follow-up was conducted 30 days after surgery using an outpatient clinic, telephone, or social media (WeChat).

**Table-I:** Comparison of intervention plans between ERAS and non-ERAS groups (traditional nursing).

Item	ERAS group	Non-ERAS group (traditional nursing)
***Before surgery***		
1. Counselling and education	The patient received education about LRC-CU at the surgical clinic, as well as information about the stoma site, which was marked by the stoma therapist.	Teaching LRC-CU-related knowledge and perioperative precautions by surgical surgeons.
2. Bowel preparation	Preoperative bowel preparation: Patients must undergo an enema the night before surgery and are required to take 400 mL of a 50 g/L glucose solution orally 2-3 hours before surgery.	Routine administration of sodium phosphate oral solution.
3. Minimise prolonged fasting	Patients are allowed clear fluids until 4 hours before the start of surgery and solids 6 hours before surgery. 500 mL (100g) carbohydrate drink 6 h before surgery (clear fast)	Patients are allowed to consume transparent liquids four hours before the start of surgery; solid food is allowed six hours before surgery.
4. Preoperative oral adjunctive analgesics	Gabapentin 300 mg night before surgery	No analgesics were used before surgery.
5. Incentive spirometry	For chest physiotherapy 1 day before surgery to be continued postoperatively	No pulmonary movement was performed before surgery.
***Intraoperative***		
1. Multimodal opioid-sparing analgesia	Intravenous injection of opioid drugs is not allowed without discussion with the attending anesthesiologist after induction.	After induction, intravenous injection of opioid drugs is routinely used.
2. Short-acting analgesia	Yes	Depends on the situation.
3. Regional anesthesia –	Combined spinal-epidural anesthesia was used.	General anesthesia is preferred.
4. Blood product transfusions as needed.	Yes	It depends on the situation.
5. Drains and NG tubes	Limit/minimise the use of drains, catheters, and NG tubes	Usually, drainage tubes and NG tubes are used.
6. Temperature management/normothermia	Intraoperative use of temperature management units (bair hugger/bair paws) for prevention of hypothermia and its consequences	No intraoperative temperature adjustment device was used.
7. Smaller incisions/extraperitoneal radical cystectomy to minimize gut handling and postoperative ileus	Extraperitoneal radical cystectomy was done in most cases	The intraperitoneal approach is used in most cases of radical cystectomy.
***After surgery***		
1. Early oral diet	Patients are encouraged to drink liquids in the evening. In order to prevent ileus, chewing gum and potassium-rich fluids are recommended after surgery. IV fluids are discontinued once adequate oral intake is achieved, usually the first morning after surgery	No such protocol was used.
2. Epidural analgesia	Patients transitioned to oral analgesics after the removal of the epidural catheter, usually 72 hours after surgery.	Epidural analgesia was not used in the postoperative period.
3. Early mobilisation	Assist the patient with limb massage starting two hours after surgery. From 2 to 6 hours after surgery, the patient’s family is instructed to help the patient change positions every hour. One day after surgery, the patient is guided to engage in moderate activity by the bedside. Starting from two days after surgery, patients were encouraged to move around in the hallway or ward.	The patient is active in the postoperative period.
4. Early stent removal	Avoid or remove the bracket as early as possible during the third POD.	Stents were removed late from the 10th to 14th postoperative period.
5. Chest physiotherapy/incentive spirometry	Continue to perform motivational lung capacity measurements after surgery.	No
6. Thromboembolic prophylaxis	Starting from POD1, wear pressure socks to prevent thromboembolism, and continue to complete the activity of getting out of bed.	Prophylaxis for thromboembolism was used only till one month of the postoperative period.
7.Bladder function exercise	After the patient is free to move around after surgery, they are guided and assisted in carrying out urinary reflexes, pelvic floor muscle contraction, compensatory urination exercises, etc.	No such protocol was used.

### Data collection:

- Baseline information, including gender, age, body mass index (BMI), educational level, marital status, lesion diameter, and disease stage.
- Perioperative indicators, including surgical time, estimated blood loss, initial exhaust time, initial bowel movement time, time to start mobilizing, and length of hospital stay.
- Hope level: evaluated based on the Herth Hope Index (HHI). HHI assesses positive readiness and expectancy (the ability of patients to maintain a positive attitude) (4-16 points), temporality and future **(**positive outlook on the future) (4-16 points), and interconnectedness (4-16 points). A higher score indicates a better hope level. HHI scoring was completed on the first day of admission, discharge, or 30 days after surgery. 3) QOL was assessed using the Assessment of Cancer Therapy-General (FACT-G), which includes functional wellbeing (comprising seven items), emotional wellbeing (six items), social/family wellbeing (seven items), and physical wellbeing (seven items). The score for each item ranges from 0 to 4 points, with higher scores indicating better QOL. QOL assessment was completed on the first day of admission, discharge, or 30 days after surgery. 4) Complications 30 days after surgery, including pyelonephritis, intestinal obstruction, incision infection, and urinary fistula.
- The incidence of readmission and death within 30 days after surgery.

### Statistical analysis:

SPSS 19.0 software program (IBM Corp., Armonk, NY, USA) was used. The Shapiro-Wilk test was used to determine age and BMI. The data normality evaluation was conducted on the following variables: lesion diameter, surgical time, intraoperative blood loss, initial exhaust time, initial defecation time, time to start getting out of bed, length of hospital stay, preoperative and postoperative HHI, and FACT-G scores. Data that conformed to a normal distribution were expressed as mean ± standard deviation. Non-normal data were expressed as median and interquartile range and analyzed using the Mann-Whitney U test. An independent sample t-test was used for inter-group comparison, and a paired t-test was used for intra-group comparison before and after the intervention. Mann Whitney *U* test was used for inter-group comparison, and the Wilcoxon signed-rank test was used for intra-group comparison before and after. The number of cases represented the count data, and the analysis was conducted using the chi-square test or Fisher’s exact probability method. *P*<0.05 indicated significance.

## RESULTS

This study included a total of 105 patients, comprising 74 males (70.48%). The age of patients ranged from 42 to 75 years, with a median age of 57 (52-62) years. There were 54 patients in the ERAS group and 51 in the non-ERAS group, with no statistically significant difference in the basic clinical characteristics between the two groups (*P*>0.05) (Table-II).

**Table-II:** Comparison of Basic Clinical Characteristics between ERAS Group and Non-ERAS Group.

Item	ERAS group (n=54)	Non-ERAS group (n=51)	χ^2^/t/Z	P
Male (yes), n (%)	36 (66.67)	38 (74.51)	0.775	0.379
Age (years)	57.80±6.45	55.98±7.28	1.35	0.178
Body Mass Index (kg/m^2^)	22.29±2.85	23.15±2.77	-1.575	0.118
***Educational level, n (%)***				
Elementary school and below	14 (25.93)	16 (31.37)	3.191	0.203
Junior high school	19 (35.19)	10 (19.61)		
High school and above	21 (38.89)	25 (49.02)		
***Marital status, n (%)***				
Married	41 (75.93)	33 (64.71)	1.587	0.208
Unmarried/divorced	13 (24.07)	18 (35.29)		
***Disease diameter (cm), M (P25/P75)***	4.15 (3.6-4.7)	3.7 (3.2-4.5)	-1.12	0.263
Disease staging, n (%)				
T_2_N_0_M_0_	33 (61.11)	36 (70.59)	1.046	0.307
T_3_N_0_M_0_	21 (38.89)	15 (29.41)		

The surgical time and estimated blood loss, as well as the times from LRC-CU to first exhaust, first defecation, first time getting out of bed, and length of hospital stay, were significantly lower in the ERAS group (P < 0.05). Table-III. On the first day of admission, there was no significant difference in any of the variables of the HHI score between the two groups (P > 0.05). After surgery, the scores of Positive Readiness and Expectancy, Temporality and Future, and Interconnectedness in both groups increased compared to before surgery, and was significantly higher in the ERAS group (*P*<0.05) (Table-IV).

**Table-III:** Comparison of perioperative indicators between the ERAS group and Non-ERAS group.

Variable	ERAS group (n=54)	Non-ERAS group (n=51)	t/Z	P
Surgical time (minutes)	412.3±58.4	451.3±67.3	-3.122	0.002
Estimated blood loss (ml)	357 (298-458)	438 (357-565)	-3.097	0.002
Initial exhaust time (hours)	30.89±5.36	34.24±5.76	-3.083	0.003
First defecation time (hours)	49.5 (43-53)	58 (50-61)	-4.872	<0.001
Start getting out of bed activity time (hours)	25 (23-27)	31 (27-35)	-5.426	<0.001
Duration of hospitalization (days)	8 (6-8)	9 (8-11)	-5.112	<0.001

**Table-IV:** Comparison of HHI Levels between ERAS and Non-ERAS groups.

Variable	ERAS group (n=54)	Non-ERAS group (n=51)	Z	P
***Before treatment***				
Positive Readiness and Expectancy	8(6-9)	8(6-9)	-0.837	0.402
Temporality and Future	8(6-9)	8(6-8)	-1.059	0.290
Interconnectedness	8(6-9)	8(6-9)	-0.795	0.427
***After treatment***				
Positive Readiness and Expectancy	13(11-14)^*^	11(9-12)^*^	-4.235	<0.001
Temporality and Future	12.5(11-14)^*^	10(8-11)^*^	-4.480	<0.001
Interconnectedness	12.5(10-14)^*^	10(8-11)^*^	-4.846	<0.001

On the first day of admission, there was no significant difference in the FACT-G scores (functional well-being, emotional status, social/family well-being, and physiological status) between the two groups, as shown in Table-V (*P*>0.05). After surgery, FACT-G scores in both groups increased compared to preoperative scores and were significantly higher in the ERAS group (P < 0.05). The incidence of complications in the ERAS group (3.70%) was significantly lower than that in the non-ERAS group (17.65%). The most common complications in both groups included pyelonephritis, intestinal obstruction, incision infection, and urinary fistula. The lower incidence of complications in the ERAS group is likely due to the comprehensive, multimodal approach that includes optimized anesthesia, early mobilization, early enteral feeding, and minimized stress response. The incidence of readmission was comparable in both groups (*P*>0.05), and no cases of mortality were reported within 30 days (Table-VI).

**Table-V:** Comparison of FACT-G between ERAS and Non-ERAS groups.

Variable	ERAS group (n=54)	Non-ERAS group (n=51)	t/Z	P
Before treatment				
Functional wellbeing	18.00±2.09	17.33±2.80	1.374	0.173
Emotional wellbeing	18(16-19)	18(17-20)	-1.710	0.087
Social/family wellbeing	16.5(15-18)	16(15-18)	-0.839	0.402
Physical wellbeing	17.09±2.46	17.75±1.95	-1.511	0.134
After treatment				
Functional wellbeing	23.81±2.59^*^	20.98±3.20^*^	5.003	<0.001
Emotional wellbeing	24(22-26)^*^	21(18-23)^*^	-4.742	<0.001
Social/family wellbeing	22(21-24)^*^	19(18-20)^*^	-6.025	<0.001
Physical wellbeing	23.09±2.46^*^	19.82±2.32^*^	7.007	<0.001

**Table-VI:** Complications and readmission within 30 days in two groups.

Variable	ERAS group (n=54)	Non-ERAS group (n=51)	χ^2^	P
Postoperative complications	2 (3.70)	9 (17.65)	5.437	0.044^b^
Pyelonephritis	1 (1.85)	2 (3.92)		
Intestinal obstruction	0 (0.00)	2 (3.92)		
Incision infection	1 (1.85)	4 (7.84)		
Urinary fistula	0 (0.00)	1 (1.96)		
Readmission 30 days after surgery	3 (5.56)	10 (19.61)	4.774	0.059^b^

***Note:*** b represents Fisher’s exact test.

## DISCUSSION

This single-center retrospective analysis used data from patients who underwent LRC-CU. Although no significant baseline differences were observed between groups, it is important to recognize that clinical variables such as age, BMI, and tumor burden can influence postoperative recovery trajectories. Ensuring baseline comparability strengthens the validity of our findings. Future studies may benefit from stratified analysis to further evaluate the impact of individual baseline variables on recovery outcomes.

The results showed that the surgical time and estimated blood loss, as well as the times from LRC-CU to first exhaust, first bowel movement, first time getting out of bed, and length of hospital stay, were significantly lower in the ERAS group compared to patients who received standard care. This finding is consistent with the research of Ashraf et al.^10^ and Lin et al.,^11^ which have all confirmed the advantages of ERAS protocols in RC combined with UDS. These early postoperative recovery indicators are not only markers of short-term recovery but may also predict long-term outcomes such as functional independence, psychological well-being, and quality of life. Previous studies suggest that earlier restoration of bowel function and mobility is associated with reduced psychological distress, improved physical function, and better long-term rehabilitation.^12^ However, this study only included patients who received the LRC-CU. Carney reported the first case of in situ ileal neobladder surgery in 1979, which is an ideal urinary diversion surgery (UDS) procedure that allows patients to restore near-physiological urinary function.^13^

However, there are multiple methods for UDS with no standard treatment plan.^14^ The selection of UDS methods should consider the patient’s physical condition, the surgeon’s experience, and the patient’s preferences.^13^–^15^ In 1963, abdominal wall stoma was first reported as a way of diverting urine flow.^15^,^16^ Currently, there are two types of ostomy methods routinely used: unilateral ostomy and bilateral ostomy. A unilateral ostomy is usually recommended to facilitate urine collection.^17^ All patients in this study underwent unilateral ostomy. Its advantages include simple operation, short surgical time, no interference with the digestive tract, and fast postoperative recovery, making it suitable for patients with relatively poor physical conditions.

The implementation of ERAS not only represents the development of a single discipline but, more importantly, the collaboration between the surgical department, anesthesiologists, and nurses.^10^,^11^ It encourages patients to participate actively in ERAS and promotes cooperation between patients and doctors in the implementation of ERAS, thereby improving the clinical outcomes of surgery.^7^–^11^ The results of this study indicate that ERAS can significantly improve perioperative indicators in LRC-CU patients and better ensure their postoperative recovery.

After UDS, patients face long-term rehabilitation care and stoma-related complications that may affect quality of life, and increase treatment costs.^3^,^5^,^18^ ERAS advocates the use of rapid and short-acting anesthesia to reduce intraoperative stress rates, accelerate recovery, promote early walking, and restore gastrointestinal motility.^10^,^11^,^19^ The results of this study showed that patients in the ERAS group had significantly higher hope level scores compared to patients who received standard care. As part of the ERAS protocol, medical staff pays more attention to communication with patients and providing psychological support. This helps to enhance and maintain close contact between patients, medical staff, and family members, further improving the level of hope.^9^,^19^

This study demonstrated that the functional well-being, emotional status, social and family well-being, and physiological status scores of the ERAS group were higher than those of the non-ERAS group. This indicates that the ERAS protocols improved multiple aspects of patients’ QOL, which is consistent with the findings of Ziegelmueller et al.^20^ and Brooks et al.^21^ In terms of physical well-being, the rapid recovery of gastrointestinal function, activity ability, and other associated factors that result from implementing ERAS protocols is highly beneficial for improving the physiological status of patients. In terms of emotional well-being, a smoother rehabilitation process would reduce anxiety and depression caused by long-term illness and slow recovery. In terms of social/family well-being, patients can return to family and social life more quickly, reducing their dependence on family care and improving their participation in social interactions. The improvement of functional well-being includes the recovery and enhancement of patients’ physical functions, daily living abilities, and other aspects.^20^,^21^

In this study, the incidence of complications within 30 days after surgery was significantly lower in the ERAS group (3.70%) compared to the non-ERAS group (17.65%). Brusasco et al.^22^ found that patients undergoing radical cystectomy who adopted ERAS protocols had significantly reduced readmission rates at 30 days and a lower incidence of complications at 90 days after surgery. A meta-analysis by Yang et al.^23^ also confirmed that adopting ERAS protocols during the perioperative period can reduce the risk of complications, such as incisional infections.^23^ However, there was no significant difference in the 30 days readmission rate between the two groups in this study. This discrepancy may be due to the bias associated with the small sample size and the nature of the research.

### Strength of the study:

This study adds to the growing body of evidence supporting the use of ERAS protocols in urologic oncology by focusing specifically on patients undergoing laparoscopic radical cystectomy combined with cutaneous ureterostomy (LRC-CU). Our findings provide meaningful insights into the role of ERAS in enhancing postoperative recovery, improving psychological well-being, and reducing complications, all of which contribute to better overall quality of life. The strengths of our study include the consistency of single-center data, the standardized implementation of the ERAS protocol, and the integrated multidisciplinary care approach. Nonetheless, the relatively small sample size and single-center design may limit the generalizability of our results. Future studies should involve larger, multicenter cohorts and longer follow-up periods to further validate the long-term impact of ERAS in this specific surgical population.

### Limitations

The relatively small sample size and single-center design may limit the generalizability of the results. Additionally, patient compliance and variations in the implementation of ERAS protocols across different healthcare settings may have introduced biases. Future studies with a larger sample size, multi-center design, and more rigorous patient selection criteria would help mitigate these limitations. Randomized controlled trials are needed to further control for potential biases and ensure that findings can be generalized across diverse populations and clinical settings.

## CONCLUSION

The application of ERAS protocols in LRC-CU not only shortens the rehabilitation process but also helps enhance psychological well-being and reduce postoperative complications. This integrated approach supports patients’ recovery across physical and emotional domains and offers practical value for improving overall outcomes in clinical practice.

### Authors’ contributions:

**ML:** Literature search, study design and manuscript writing. Manuscript revision and validation and is responsible for the integrity of the study.

**WC, TC, LL, HF and KL:** Data collection, data analysis and interpretation. Critical analysis.

All authors have read and approved the final manuscript.
